# Editorial: Methods in alloimmunity and transplantation: 2025

**DOI:** 10.3389/fimmu.2026.1970388

**Published:** 2026-08-31

**Authors:** Martin Kauke-Navarro, Guido Moll

**Affiliations:** 1 Department of Surgery, Division of Plastic and Reconstructive Surgery, Yale New Haven Hospital, Yale School of Medicine, New Haven, CT, United States; 2 BIH Center for Regenerative Therapies (BCRT) and Berlin-Brandenburg School for Regenerative Therapies (BSRT), Charité, Berlin, Germany; 3 Julius Wolff Institute (JWI) for Musculoskeletal Research, Charité, Berlin, Germany; 4 Department of Nephrology and Internal Intensive Care Medicine, All Three Part of Charité Universitätsmedizin Berlin, Corporate Member of Freie Universität Berlin, Humboldt-Universität zu Berlin, and Berlin Institute of Health (BIH), Berlin, Germany

**Keywords:** alloimmunity, donor-derived cell-free DNA, HLA and non-HLA antibodies, immunosuppression, inflammation, non-invasive biomarkers, rejection, transplantation

## Introduction

**Figure 1:**
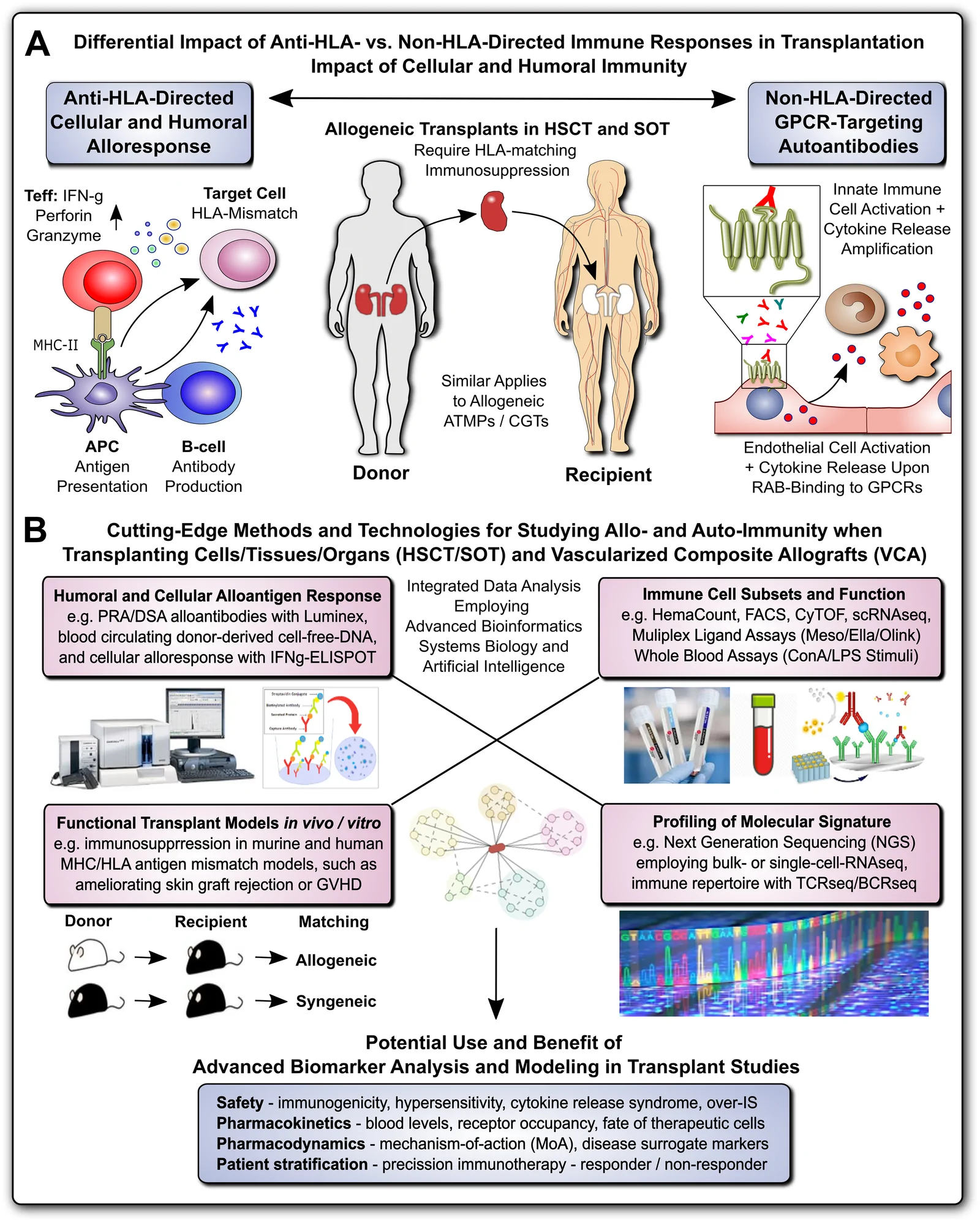
Methods for studying allo- and auto-immunity in transplantation and autoimmunity (2025 version adapted from the 2023 version) (1). **(A)** Differential Impact of Anti-HLA- and Non-HLA-directed Immune Responses in Transplantation: Allogeneic transplants in HSCT and SOT typically require HLA-matching and immunosuppression to prevent allograft rejection through anti-HLA-directed alloantigen-specific immune responses (e.g. T and B cell and alloantibody mediated), with a minor but significant contribution from non-HLA-directed auto-antigen-specific autoantibodies (e.g. GPCR-directed regulatory autoantibodies, RABs) (2, 9, 10). **(B)** Cutting-Edge Methods and Technologies for Studying Allo- and Auto-Immunity when Transplanting Cells/Tissues/Organs and Vascularized Composite Allografts (VACs): entailing at least four major important categories, such as detailed studies of: 1) Humoral and Cellular Alloantigen Responses, including monitoring of PRA/DSA with Luminex, blood circulating dd-cfDNA, and detection of cellular alloresponses with ELISpot typically IFNg-specific; 2) Functional Transplant Models *in vitro/in vivo*, including the study of novel immunosuppressive drugs and drug regimens in murine and human tissue MHC/HLA antigenic mismatch models, such as ameliorating allogeneic skin-graft rejection in mice or ameliorating GVHD in the HSCT setting; 3) Immune Cell Subsets and Function, including the use of hematology counters for absolute and relative cell quantification in whole blood, and targeted multiparametric analysis with flow cytometry/FACS and CyTOF with pre-defined panels, or broad-scale scRNAseq analysis for unbiased analysis of highly diverse cellular subsets, but also various multi-ligand-plex systems, (e.g. Mesoscale, Ella, and Olink) with different levels of sensitivity for specific ligands, and in addition whole blood assays (e.g. employing LPS or ConA stimulation for differential readout of cell type specific immune responses); and 4) Global Profiling of Molecular Signatures, including NGS-based analysis of bulk transcriptome with conventional RNAseq technology or at single-cell level with scRNAseq, and immune cell repertoire with TCRseq and BCRseq. In particular the integrated analysis of data from different analysis/modeling/readout platforms with advanced bioinformatics, including systems biology and artificial intelligence is of interest for optimal data interpretation and identification of suitable biomarkers. The potential use and benefit of advanced biomarker analysis and modeling in transplant studies entails multiple aspects, including: 1) Safety Assessment: such as immunogenicity, hypersensitivity, cytokine release syndrome, or over-immunosuppression (IS); 2) Pharmacokinetics: such as blood levels and receptor occupancy of specific ligands, or the fate of therapeutic cells; 3) Pharmacodynamics: such as studies on the mechanisms-of-action (MoA) and disease-specific surrogate markers; and 4) Patient Stratification: enabling precision immunotherapy by better understanding and distinguishing or restratifying responder and non-responder patients in advanced clinical trials.

This Research Topic is the second volume of the “Methods in Alloimmunity and Transplantation” series and part of the wider “Methods in Immunology” series, in which recent developments and methodological topics are discussed (**Figure 1**) (1–3). Alloimmunity describes the immune response to alloantigens: immunogenic molecules from other members of the same species, typically blood group antigens and the highly polymorphic major histocompatibility complex (MHC) antigens (1–6). Alloantigens are traditionally divided into major and minor mismatch antigens, and they are distinguished from the xenoantigens of other species and from the autoantigens or self-antigens (1–16). They engage both arms of the adaptive response: alloantibody formation through primed B cells and plasma cells, measured as panel-reactive and donor-specific antibodies and autoantibodies (PRA and DSA and AABs), but also in the activation/polarization of effector and regulatory T cells (Teffs and Tregs) (1–16). The clinical modalities that depend on controlling this response include hematopoietic stem cell transplantation (HSCT), solid organ transplantation (SOT; e.g. kidney, liver, lung and heart), and vascularized composite allotransplantation (VCA) (1–3, 17–27). In VCA, face allografts carry a large and highly immunogenic mucosal and skin component which makes strict rejection monitoring even more important and adds another layer of complexity (20–27).

Indeed, across all the transplant (Tx) procedures, outcomes still depend on two major variables: how precisely donor and recipient can be matched, and how narrowly immunosuppression (IS) can be targeted, without trading rejection for infection, malignancy, and drug toxicity (20–22, 28–30). The first volume of this Research Topic has described several established and emerging methods (**Figure 1**), which have now moved much closer to routine, including allo/auto-antibody-profiling with Luminex and other technologies (e.g. whole autoantibodyome assessment with HuProt-Assays), next-generation-sequencing (NGS)-based methods (e.g. RNAseq and single-cell RNAseq (scRNAseq), T- and B-cell receptor repertoire sequencing (TCRseq and BCRseq) (31–33), and NGS analysis of donor-derived cell-free DNA (dd-cfDNA) (34–40). Importantly, dd-cfDNA is now increasingly used for surveillance rather than as a research readout in a growing number of transplant centers (41). However, the timing and ideal detection thresholds and fractional versus absolute quantification across different organ types are all still under investigation (e.g. KTx vs LuTx) (35). Molecular diagnostics on biopsy tissue increasingly run alongside Banff histopathology (39, 42). Single-cell sequencing, spatial transcriptomics, and artificial intelligence and machine learning (AI/ML)-assisted systems biology (SysBio)-analysis now generate comprehensive readouts, where the data interpretation and not the acquisition is the hardest part (14, 42–47). In the fields of transplantation, allo- and auto-immune diagnostics, technologies are now moving forward from monitoring individual molecular markers and determinants, to assessing whole transcriptome and larger allo/auto-antibody-panels in combination with scRNAseq profiling of cellular composition, to complement the traditional Banff criteria and to better understand the whole picture of acute and chronic rejection processes and autoimmunepathology in individual patients and larger cohorts (10, 11, 42–44, 48–53).

In clinical practice, novel concepts of transplantation (Tx), immunosuppression (IS), cell therapy and patient care are moving rapidly forward (28, 29). This includes therapy with advanced therapy medicinal products and cell and gene therapies (ATMPs and CGTs), such as immunomodulatory and regenerative mesenchymal stromal/stem cells (MSCs) and Tregs to improve graft tolerization and ameliorate endogenous rejection processes, but also CD19-directed chimeric antigen receptor (CAR)-modified immune cell therapy for depletion of pathogenic allo/auto-antibody-producing B- and plasma-cell memory (2, 15, 17, 29, 45, 46, 54–63). Two promising recent case studies from independent centers have shown that depletion of anti-HLA antibodies with CD19-directed CAR-T cell therapy can facilitate KTx in patients previously at risk of rejection due to high allo-antibody sensitization (15, 16, 64). Similar applies in the treatment of autoimmunepathology and oncology, where CAR-T therapy has first emerged approximately one decade ago as promising new treatment modality for cancer, but now also realizing its enormous potential for the treatment of autoimmune and transplant pathology (65).

The eighteen contributions gathered in this Research Topic fall into four groups: non-invasive and tissue-based diagnostics of rejection; experimental and clinical models of alloimmune and complement-mediated injury; the humoral and cellular mechanisms these methods aim to capture; and cell- and gene-based therapeutics together with their clinical outcomes.

## Non-invasive and tissue-based diagnostics of rejection

The largest cluster in this volume addresses a problem the field has not yet solved: how to detect allograft injury early and non-invasive - without the need for repeated biopsies. Barr et al. have surveyed a number of non-invasive biomarkers, which are near or already in clinical use for kidney allografts — dd-cfDNA, blood gene-expression profiling, urinary CXCL9/CXCL10 chemokines, extracellular vesicles, microRNA. Their central message is a methodological caution: most of these markers have strong negative predictive value for ruling rejection out but modest positive predictive value. Thus, they nowadays still complement rather than replace conventional histology.

Two studies extend the assessment of dd-cfDNA beyond the kidney. Deng et al. have evaluated dd-cfDNA as a non-invasive marker for diagnosing and monitoring acute rejection after liver transplantation. Furthermore, Calabrese et al. report a real-world single-center experience measuring dd-cfDNA in both plasma and bronchoalveolar lavage to detect lung allograft injury. Intriguingly, the authors show that the compartment sampled matters as much, as the quality of the assay itself. Both show, how a kidney-validated readout behaves differently, once moved to organs with other biology and other background cfDNA.

In cytomegalovirus (CMV)-monitoring, Casas-Parra et al. directly compared four assays for CMV-specific cell-mediated immunity in solid organ recipients. Barison et al. offer a proof-of-concept for transcriptomic profiling of CMV infection in cardiac transplantation as a tissue-marker strategy. Moving from viral to alloimmune diagnostics, Kauke-Navarro et al. have compared lesional skin, sentinel-flap, and mucosal biopsies for assessing acute rejection in face transplantation, quantifying how different biopsy site can influence the histological picture in VCA (22). The authors describe a discordance with direct consequences for how rejection is graded and treated. These findings matter for VCA and build on earlier reports: skin and mucosa reject with distinct spatial and temporal patterns and through different mechanisms. B cells, for example, are found in mucosa but not skin, and the discordance between skin and mucosal grades widens as the mucosal rejection grade rises (23–27). This is in line with other studies listed below that clearly outline immunological differences in tissue compartments and how they are affected by the host’s immune system.

Finally, two contributions apply modelling and synthesis to the diagnostic problem. Mahler et al. develop and validate data-driven and expert-based prediction models for evaluating deceased-donor kidney offers and early transplant outcomes, and Danso et al. provide a systematic review of rejection mechanisms, monitoring, and immunosuppressive therapeutics across solid organ transplants, giving the collection a broad reference point.

## Models and mechanisms of alloimmune and complement-mediated injury

Understanding the signals these diagnostics detect requires controlled experimental systems and detailed mechanistic work. In VCA, Thomé et al. establish a rodent model that distinguishes acute from chronic skin rejection. Schweizer et al. use fully mismatched rat hind-limb transplants to test donor adipose-derived mesenchymal stromal/stem cells (MSCs) against FK-506, finding that the MSCs partially delayed, but did not reverse progression from grade II to grade III rejection. The authors demonstrate that the agent was able to reduce graft vasculitis and pro-inflammatory cytokines. That negative result on rejection reversal is as informative as the vasoprotective one: it argues that stromal cell therapy in VCA might be an adjunct to conventional immunosuppression, and not a replacement.

On the mechanistic side, She et al. review how neutrophils contribute to ischemia-reperfusion injury and rejection in liver transplantation, and Pollmann et al. report complement-mediated thrombotic microangiopathy after liver transplantation in a recipient carrying a novel C6 variant of uncertain significance, a detailed clinical-immunological case linking a specific complement lesion to a post-transplant complication. The same group’s systematic review and meta-analysis quantifies the worldwide impact of recent COVID-19 infection on liver and kidney transplantation. They address a methodological question: how peri-transplant infection should weigh on timing decisions.

## Humoral and cellular alloresponses

Two contributions dissect the humoral mechanisms some of these diagnostics are ultimately trying to capture. Mizera et al. show that anti-AT1R autoantibodies of the IgG3 subclass outperform total anti-AT1R IgG1–IgG4 levels in predicting antibody-mediated rejection of kidney allografts. Liu et al. review B cell-mediated immune reconstitution after lung transplantation, arguing that donor-specific antibodies and chronic lung allograft dysfunction are best understood through stage-specific B cell dynamics. The authors point out that single-cell RNA sequencing and B-cell receptor repertoire profiling are the tools that could bring that resolution into the clinic.

## Cell and gene therapy and clinical outcomes

The final group reports on therapeutics and their outcomes. Trevisan et al. show in a pediatric sheep model that transplanted gene-modified placental cells boost factor VIII activity without eliciting immunity, toxicity, or adverse events. This study presents a large-animal proof-of-concept for gene-modified cell therapy that directly addresses the immunogenicity questions such products must clear. Two clinical HSCT studies close the collection: Ringdén et al. report long-term survival in three pancreatic cancer patients who received allogeneic HSCT after Whipple’s procedure, an unusual observation of a graft-versus-tumor effect in a solid malignancy, and Hao et al. present a single-center study of allogeneic HSCT for mixed-phenotype acute leukemia, a rare and difficult-to-treat entity where transplant outcome data remain scarce.

## Outlook

The methodological direction across these eighteen contributions is consistent: away from single-analyte, single-time-point diagnostics and toward composite monitoring that combines a molecular signal, an antibody/autoantibody/autoantibodyome profile, and a cellular readout in the same patient over time. Two obstacles become clear. Assays originally validated in kidney transplantation are being transferred to liver, lung, heart, and VCA without re-devised thresholds. Multi-center standardization and comparable data-integration still lags well behind analytical innovations and single-center advancements, thus calling for continued cooperation towards future advancements. Novel treatment concepts and cell therapy approaches now offer safe and effective new treatment modalities for pathology previously refractory to treatment. We thank the authors, reviewers, and the Frontiers editorial office, and we hope the next volume can report on how far that standardization has come.
